# Pulmonary Tuberculosis and a Related Verrucous Plaque on the Sole of the Foot

**DOI:** 10.4269/ajtmh.26-0301

**Published:** 2026-09-15

**Authors:** Akshay Meena, Saka Sowmya Susan, Sreerekha Jinkala, Sheetanshu Kumar

**Affiliations:** ^1^ Dermatology and STD, Jawaharlal Institute of Postgraduate Medical Education & Research, Pondicherry, India;; ^2^ Department of Pathology, Jawaharlal Institute of Postgraduate Medical Education & Research, Pondicherry, India

**Figure 1.**
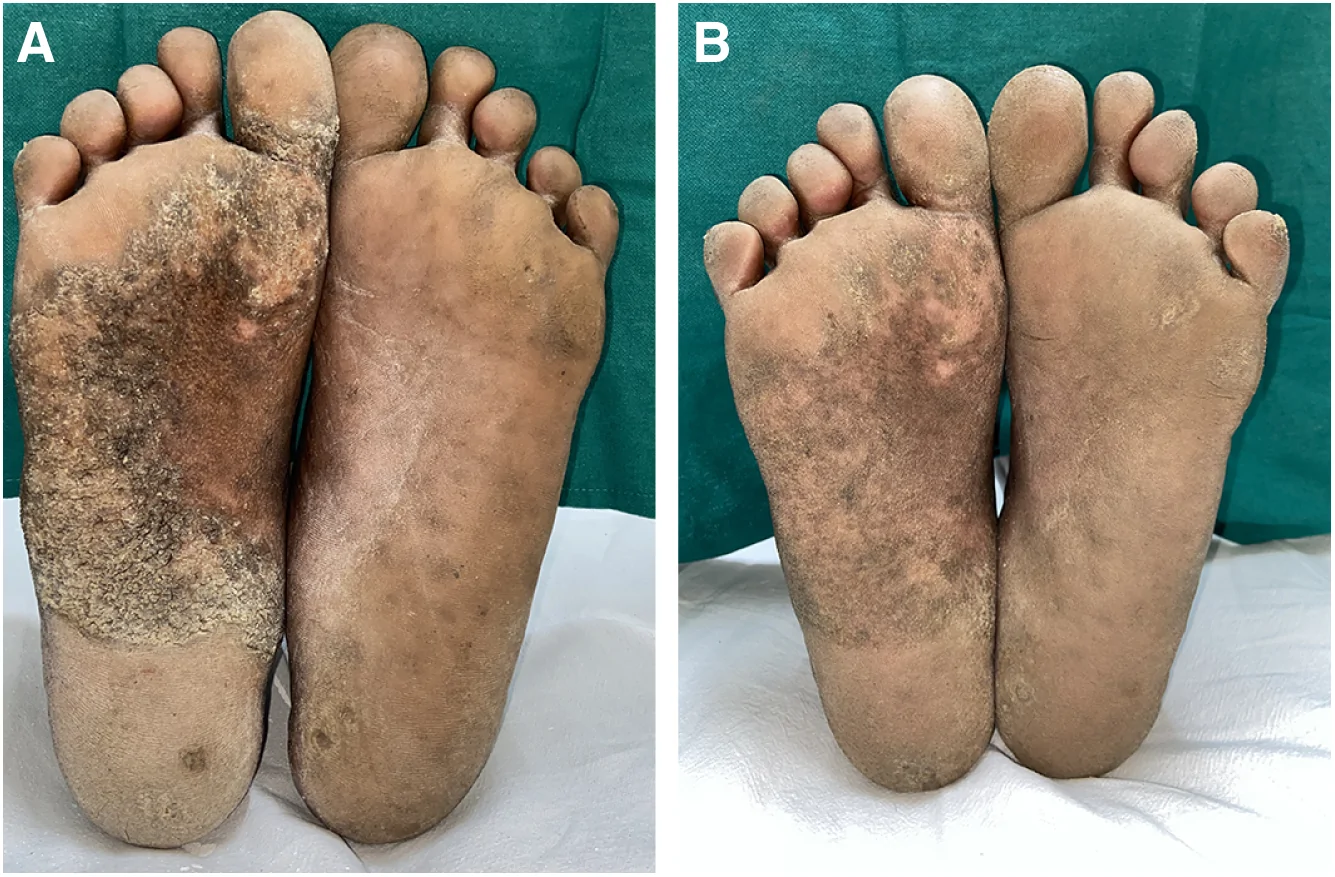
(**A**) Single well-defined hyperkeratotic plaque with verrucous surface measuring approximately 10 × 8 centimeters over the right sole. (**B**) Significant improvement at the follow-up visit.

**Figure 2.**
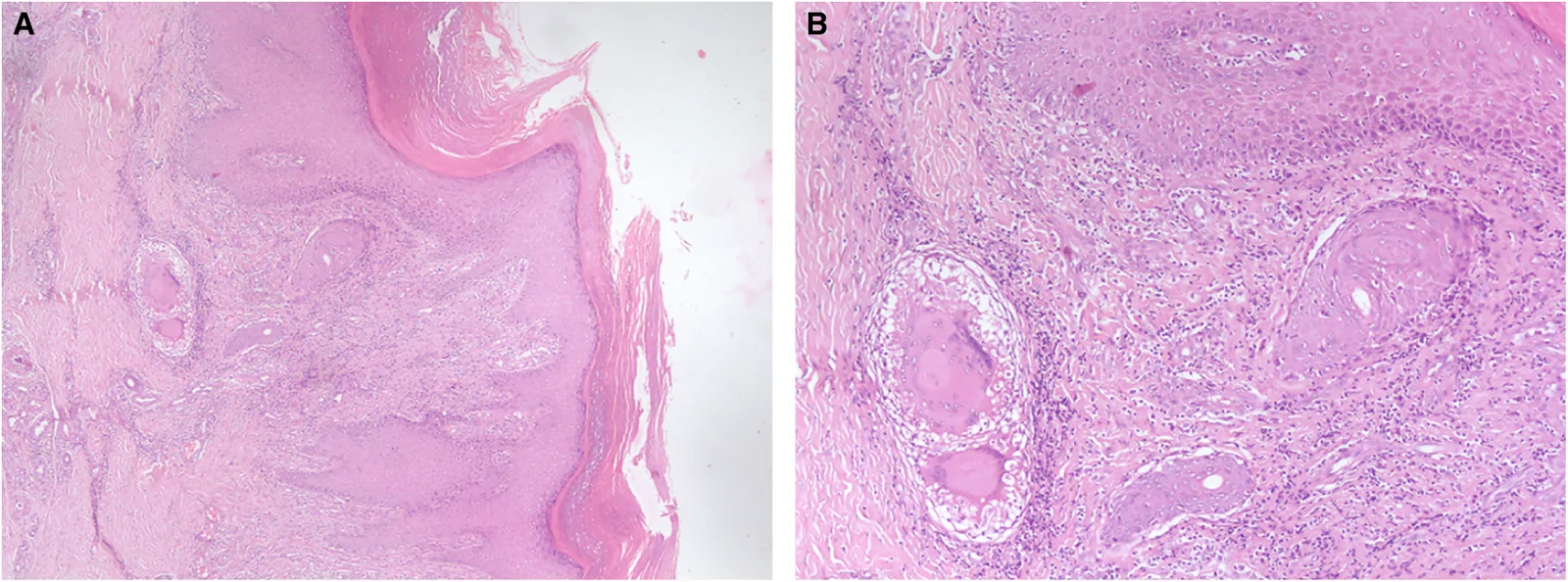
(**A**) Histopathological examination showed marked hyperkeratosis and acanthosis with pseudoepitheliomatous epidermal hyperplasia and granulomatous inflammatory infiltrate in the dermis (hematoxylin and eosin, ×4). (**B**) Higher power showed tuberculoid granuloma composed of epithelioid histiocytes with surrounding lymphocytic infiltrate (hematoxylin and eosin, ×10).

**Figure 3.**
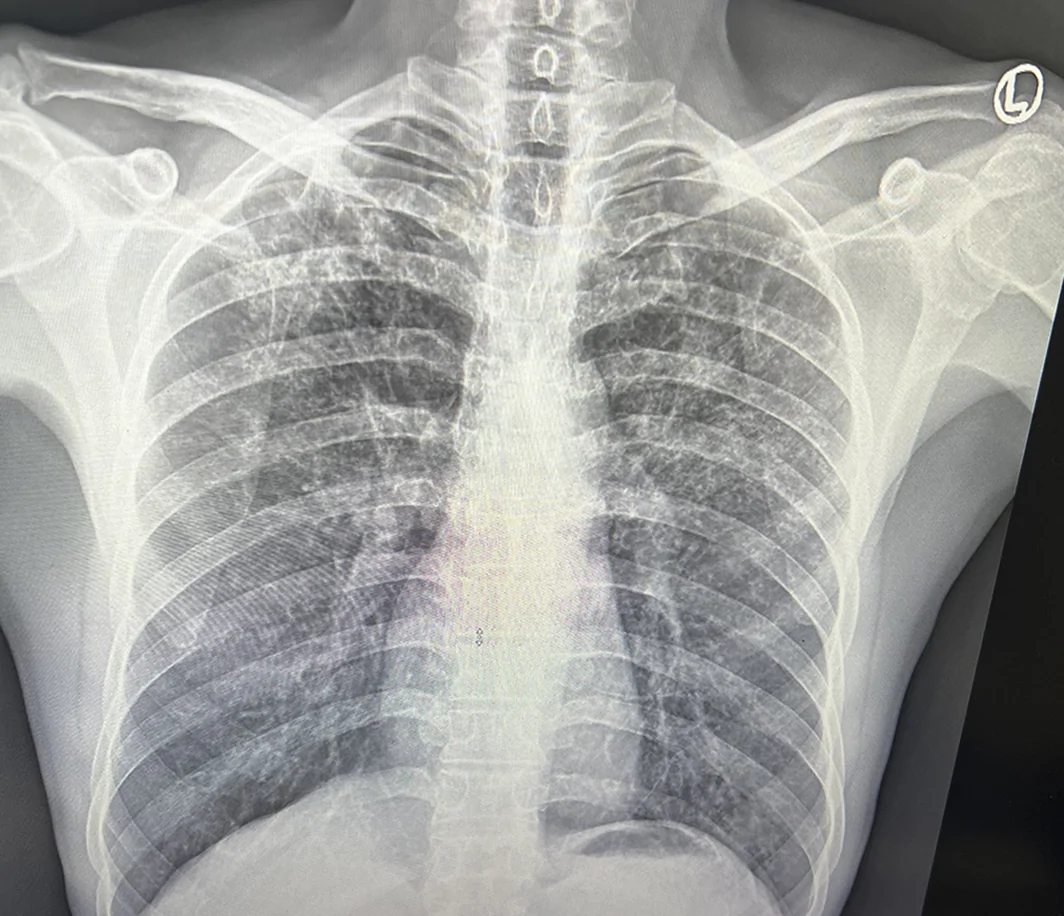
Chest radiograph showing bilateral patchy perihilar and interstitial infiltrates involving the middle and lower lung zones.

A 50-year-old man presented with a 5-year history of progressively enlarging pruritic verrucous plaque on the plantar aspect of the right foot. He denied any history of trauma, fever, night sweats, weight loss, cough, or shortness of breath. He reported walking barefoot. Cutaneous examination revealed a solitary, well-defined hyperkeratotic plaque with a verrucous surface measuring approximately 10 × 8 centimeters with no evidence of central healing or scarring (Figure 1A). The rest of the skin, mucosa, nails, and systemic examination were unremarkable, and there was no regional lymphadenopathy. An incisional biopsy was done with the differential diagnosis of plantar psoriasis, plantar lichen planus, chromoblastomycosis, tuberculosis verrucosa cutis (TBVC), and atypical mycobacterial infection. Histopathological examination showed marked hyperkeratosis, hypergranulosis, and acanthosis along with focal basal vacuolar degeneration. The upper dermis showed well-formed epithelioid granulomas with multinucleated giant cells along with a moderate perivascular and periappendageal lymphohistiocytic infiltrate (Figure 2). Ziehl–Neelsen staining for acid-fast bacilli was negative. Chest radiography revealed bilateral patchy perihilar and interstitial infiltrates involving the middle and lower lung zones without focal lobar consolidation (Figure 3). GeneXpert analysis of induced sputum detected *Mycobacterium tuberculosis* with no evidence of rifampicin resistance. Routine laboratory investigations were normal, and HIV serology was negative. Based on clinicopathological correlation, a diagnosis of TBVC was inferred, and the patient was started on antitubercular therapy consisting of isoniazid, rifampicin, pyrazinamide, and ethambutol in accordance with national tuberculosis treatment guidelines. The patient showed significant improvement in cutaneous and pulmonary disease at the 3-month follow-up visit (Figure 1B).

Tuberculosis verrucosa cutis is a paucibacillary form of cutaneous tuberculosis resulting from exogenous reinfection in previously sensitized individuals. In an Indian study, lupus vulgaris was the most common variant, whereas TBVC accounted for nearly 5% of cases.^1^ It commonly occurs in tropical countries and often affects individuals who walk barefoot, with the lower extremities being the usual site of involvement.^2^ Psoriasiform, keloidal, crusted, exudative, sporotrichoid, destructive, and tumor-like forms are the main variants of TBVC. Clinically, TBVC evolves from a small papule into a slowly enlarging, indurated, warty plaque with fissures and centrifugal growth, closely mimicking conditions such as verruca vulgaris, chromoblastomycosis, hypertrophic lichen planus, and palmoplantar keratoderma.^3^ Histology typically shows pseudoepitheliomatous hyperplasia and granulomatous inflammation, whereas acid-fast bacilli are rarely detected. Molecular tests, such as GeneXpert, significantly improve diagnostic yield. Dermoscopy may reveal thick scales, papillated surfaces, and yellow globules corresponding to granulomas.^4^

This case highlights the skin as a sentinel organ.^5^ The coexistence of pulmonary tuberculosis and TBVC in our patient raises interesting pathogenetic considerations. Tuberculosis verrucosa cutis is traditionally attributed to direct exogenous inoculation of *M. tuberculosis* into previously sensitized skin, particularly at trauma-prone sites, such as the sole. However, the presence of clinically silent pulmonary tuberculosis raises the possibility that the cutaneous lesion may represent an exaggerated immune response to occult systemic infection, analogous to a tuberculid. Nevertheless, the long-standing verrucous plaque and the characteristic histopathological findings favor true cutaneous tuberculosis rather than a classical tuberculid. The precise temporal sequence between pulmonary and cutaneous involvement, therefore, remains speculative. A long-standing plantar lesion, which was initially overlooked, led to the diagnosis of clinically silent pulmonary tuberculosis. Thus, confirmation of TBVC should prompt evaluation for systemic involvement, particularly in endemic regions, as cutaneous findings may precede or reveal otherwise occult internal disease.
